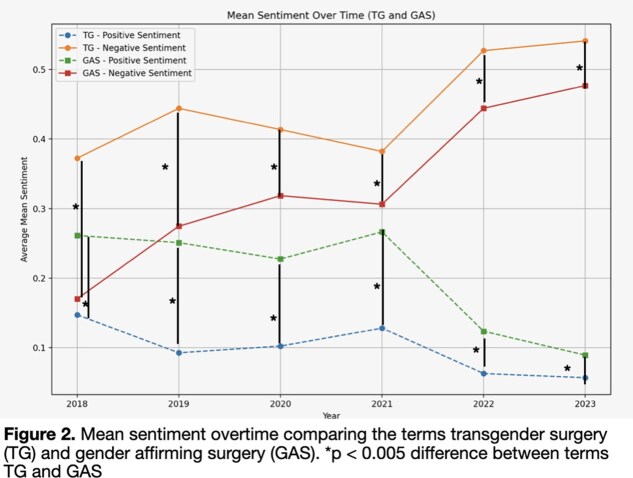# The Shifting Tides of Sentiment: A 5-Year Analysis of Gender Affirming Surgery Discourse on Social Media

**DOI:** 10.1093/asjof/ojaf018.008

**Published:** 2025-05-13

**Authors:** Cole Holan, Arman Fijany, Jorys Martinez-Jorge

**Affiliations:** Mayo Clinic, Rochester, MN; Vanderbilt University Medical Center, Nashville, TN; Mayo Clinic, Rochester, MN

## Abstract

**Goals/Purpose:**

This study aims to examine the sentiment and polarization of social media content regarding transgender care. We seek to quantitatively analyze social media posts related to transgender care using sentiment analysis, assessing the extent of positive or negative bias in online discussions about transgender healthcare. By establishing a baseline understanding of how online misinformation may impact public perceptions of transgender care, we highlight the importance of addressing online extremism in healthcare as a public health concern. This research represents the first large-scale natural language processing (NLP) analysis of online sentiment towards transgender care, utilizing sentiment analysis to minimize bias and provide quantitative insights into the impact of online rhetoric on public perception and patient care.

**Methods/Technique:**

The lead author mined X for posts that mentioned transgender surgery (TG) and gender affirming surgery (GAS) from January 2018 to December 2023. Two different NLP models then analyzed the posts. The first was a binary model that characterized each post as positive or negative. The latter was an emotion-based model that scored each post about seven emotions – fear, sadness, anger, disgust, neutral, surprise, and joy. Posts were then classified by their highest-scoring emotion. An exponential regression analysis within the Python language was performed to study the trend in annual X post volume related to gender surgery. To determine whether there were statistically significant differences in sentiment between transgender (TG) and gender-affirming surgery (GAS) topics, independent two-sample t-tests were conducted for both positive and negative sentiments for each year within the study period.

**Results/Complications:**

In total, there were 81,264 posts related to gender surgery included in our analysis. In 2018 there were 2,093 posts, compared to 2023 where there were 40,258 posts – a roughly 20-fold increase in annual post volume. In our exponential regression model, annual post volume demonstrated a strong positive trend R2 = 0.939, p = 0.001, Figure 1). Most of the posts were classified as ‘negative’ (63,192 posts, 78%). Mean scores for both GAS and TG posts trended towards more negative and less positive sentiments over the study period (Figure 2). When comparing annual TG to GAS posts, those that mentioned TG were significantly more negative and less positive in every year studied (p < 0.05).

**Conclusion:**

Our analysis of social media posts related to gender-affirming surgery (GAS) and transgender (TG) care reveals a significant increase in online discourse over the past five years. The volume of posts grew exponentially from 2,093 in 2018 to 40,258 in 2023, representing a 20-fold increase. This rise in online discussion reflects the growing public interest and controversy surrounding transgender healthcare.The sentiment analysis revealed a trend toward increasingly negative rhetoric. A substantial majority (78%) of the posts were classified as negative, indicating a prevalence of critical or hostile attitudes towards GAS and TG care on social media platforms. The mean sentiment scores for both GAS and TG-related posts showed a consistent shift toward more negative and less positive sentiments over time. Notably, posts specifically mentioning transgender individuals or issues consistently displayed more negative and less positive sentiments compared to general GAS-related posts across all years studied. This finding suggests that transgender-specific topics are subject to particularly intense scrutiny and criticism online.These results highlight the urgent need for strategies to address misinformation and negative rhetoric surrounding transgender healthcare on social media platforms. The increasing volume and negativity of online discussions may contribute to a hostile environment for transgender individuals seeking care and for healthcare providers offering these services. Future research should focus on developing effective interventions to counter this trend and promote more balanced, informed discussions of transgender healthcare issues online.

**Figure:**
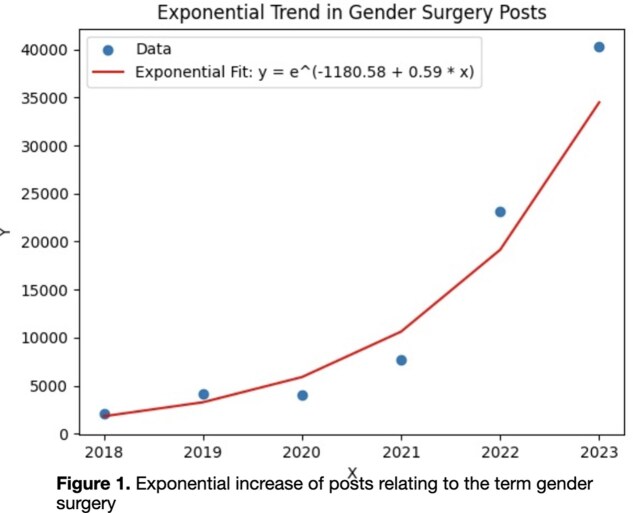

**Figure:**